# Maternal Distress and Adolescent Mental Health in Poor Chinese Single-Mother Families: Filial Responsibilities—Risks or Buffers?

**DOI:** 10.3390/ijerph20075363

**Published:** 2023-04-03

**Authors:** Janet T. Y. Leung, Daniel T. L. Shek, Siu-Ming To, So-Wa Ngai

**Affiliations:** 1 Department of Applied Social Sciences, The Hong Kong Polytechnic University, Hong Kong; 2 Department of Social Work, The Chinese University of Hong Kong, Hong Kong

**Keywords:** filial responsibility, maternal distress, adolescent mental health, single-mother families, poverty, Chinese

## Abstract

Single motherhood and poverty have a significant, negative impact on mothers and their children. When their mothers experience maternal distress, adolescent children have to take up more instrumental and emotional filial responsibilities to comfort their mother and adapt to related changes. Based on 325 mother–child dyads of Chinese single-mother families experiencing economic disadvantage, this study examined the relationship between maternal distress and adolescent mental health problems (indexed by anxiety and depression) and the moderating roles of instrumental and emotional filial responsibilities. Results indicated that maternal distress was positively associated with anxiety and depression in adolescent children. In addition, instrumental filial responsibility intensified the associations of maternal distress with adolescent anxiety and depression. Moreover, the moderating role of emotional filial responsibility in the predictive relationship between maternal distress and adolescent anxiety was different in boys and girls. Adolescent girls with more emotional filial responsibility reported higher adolescent anxiety than did those who shouldered less emotional filial responsibility when their mother exhibited more distress, whereas the relationship between maternal distress and adolescent anxiety was stable in boys, regardless of emotional filial responsibility. In short, the present study showed that parentification was likely to occur in poor Chinese single-mother families, and adolescent children who took up a more caregiving role in the family exhibited poorer mental health. Family counselling and tangible support for single-mother families experiencing economic disadvantage are urged.

## 1. Introduction

As divorce rates in many countries have rapidly increased over the past few decades [1], single parenthood has become a social issue in global and local contexts. In mainland China, the crude divorce rate (i.e., the number of divorce decrees granted per 1000 persons) had increased dramatically in the past ten years from 1.85 in 2009 to 3.36 in 2019 [2], pushing the State to enforce a 30-day cooling-off period for divorce seekers in 2021. Hong Kong could not escape from the global trend. The crude divorce rate in Hong Kong rose from 1.11 in 1991 to 2.82 in 2019, with a drop to 2.14 in 2020 due to service lockdown because of the COVID-19 pandemic [3]. The proportion of single parent population to overall population of parents with children under 18 years old increased from 3.9% in 2001 to 5.9% in 2016. Among them, 78% are single mothers [4].

Research studies have showed that single mothers are vulnerable to poverty. They face more difficulties for open employment due to child-rearing demand [5]. Even if they are available for the job market, their work stability, as well as salary are, relatively, lower than those of single fathers [6]. In 2016, the median monthly household income of single-mother families in Hong Kong was HKD 13,780 (USD 1778.2), while the corresponding figure was HKD 18,000 (USD 2307.7) in single-father families and HKD 25,000 (USD 3205.1) in Hong Kong as a whole [4]. Obviously, single-mother families are at the edge of the poverty threshold (i.e., HKD 12,500 (USD 1602.6)). The absence of a husband and concurrent poverty further create vulnerabilities to single mothers. Chant [7] used the term “feminisation of poverty” to describe single mothers who are prone to poverty and face great challenges to uplift their families out of poverty.

### 1.1. Maternal Distress and Adolescent Mental Health

The Family Stress Model accounts for the relationship between maternal distress and adolescent internalization and externalization of problems among economically disadvantaged families [8]. In the context of poverty, parents feel stressed when they face economic hardship. Debts and material deprivation bring burdens to parents, which are associated with distress and anxiety [9,10,11]. The experience is particularly stressful for single mothers, when they exhibit additional negative emotions of depression, resentment and abandonment due to the loss of the spousal relationship [12]. Jones et al. [13] identified several risk factors in single-mother families, including inadequate income, maternal depression and disruptive parenting. As the negative emotions of single mothers would spill over to their children, there is evidence showing that maternal depression was associated with poorer mental health in their offspring [14,15].

### 1.2. Adolescent Filial Responsibility as a Moderator

To support mothers in household management and restore family functioning, adolescent children usually take up more responsibilities in the family [16]. Filial responsibility is the children’s effort to give assistance and support to the family [17,18]. Typically, there are two types of filial responsibilities: instrumental and emotional. While instrumental filial responsibility refers to the fulfilment of household chores and daily caregiving for the siblings, emotional filial responsibility is the support for the emotional needs of parents and siblings [19]. Due to scarce resources, adolescents need to share the household chores at home and act as the “surrogate parents” of the younger siblings [20]. Moreover, they may pick up the “junior-partner” role to comfort their mother’s emotions and act as their mother’s confidants [19]. As such, filial responsibility may moderate the impact of maternal distress on adolescent mental health.

In accounting for the moderating roles of filial responsibility in altering the relationship between maternal distress and adolescent mental health problems, both Family Systems Theory [21,22] and Role Identity Theory [23,24] provide diverse answers to this issue. Parentification has long been studied as a clinical phenomenon that describes a process in which a child assumes a developmentally inappropriate parental role to perform family responsibilities [22,25,26]. Parentification is commonly identified in single-parent families (e.g., [19]) and poor families [27,28] when parents face more difficulties in managing life and family stresses. Thus, their adolescent children are obligated to support the family to maintain proper family functioning. According to Family Systems Theory, parentification involves role reversal that reduces or blurs the intergenerational boundaries, and the enmeshed relationship suppresses the age-appropriate needs of the children as they need to fulfil the demands of their parents and siblings, which is harmful to their development [22]. In summary, the parentified children are described as “burdened children” [26] who have lost their childhood [29]. Parentification was found to be linked to adolescent pathologies such as depression, somatic symptoms, excessive guilt, etc. [26,30].

However, according to Social Identity Theory [31], family responsibilities are considered normative and necessary for building family solidarity and stability, particularly among collectivist societies [23,24]. Under this framework, filial responsibilities promote adolescent positive psychosocial outcomes by demonstrating family devotion and establishing connectedness with family members. Indeed, culture plays an indispensable role in shaping the ecology of family socialization and adolescent development [32]. In Chinese societies where collectivism, familism and interdependence are emphasized [33], filial piety has become the cardinal rule for intergeneration conduct, which dictates family obligations of the children to their parents in the Chinese ethical system [34]. Children are socialized to provide material and psychological support to parents, fulfil the parental wishes, bring pride to the family, and avoid disgrace to the family name [35]. In a study of 432 poor single-mother families in Hong Kong, Leung and Shek [36] found that higher filial piety was associated with better adolescent development. Filial responsibility was also positively linked to parent–child cohesion [17], which may reduce maternal distress [37]. From this view, filial responsibility may serve as a buffer that smooths the association of maternal distress with adolescent mental health problems.

### 1.3. Moderation of Adolescent Gender and Age

Typically, girls take up more instrumental and emotional caregiving than do boys [38], and girls are more sensitive to affective bonding with mothers than boys [39]. Hence, it is expected that the influence of filial responsibilities on adolescent wellbeing and mother–child relationships will be stronger in girls than boys. Unfortunately, the findings in this area are inconclusive. While some studies showed that girls were more negatively affected in taking up the caregiving roles (e.g., [40]), other studies did not find a significant difference across adolescent gender (e.g., [41]).

Moreover, older adolescents request greater autonomy and more space for personal development and social engagement when they grow up [20,42]. In addition, they are more likely to take up a larger share of household chores than are their younger siblings [43]. Hence, compared to younger siblings, older siblings may feel disturbed and annoyed when they spend much of their time and effort in taking care of their family [42], which may lead to distress.

### 1.4. The Present Study

The present study examined the moderating roles of instrumental and emotional filial responsibilities in the relationship between maternal distress and children’s mental health (indexed by anxiety and depression) among Chinese single-mother families experiencing economic disadvantage. There are several research questions:

Research Question 1: What is the relationship between maternal distress and children’s mental health (indexed by anxiety and depression) among Chinese single-mother families experiencing economic disadvantage?

**Hypotheses** **1.** *It was hypothesized that maternal distress would be positively associated with anxiety (H1a) and depression (H1b) in adolescent children among Chinese single-mother families experiencing economic disadvantage.*

Research Question 2: Do instrumental and emotional filial responsibilities moderate the relationship between maternal distress and children’s mental health (indexed by anxiety and depression) among Chinese single-mother families experiencing economic disadvantage?

**Hypotheses** **2a and 2b.** *Relative to a lower level of instrumental filial responsibility, the relationship between maternal distress and adolescent anxiety (H2a) and depression (H2b) would be higher under a higher level of instrumental filial responsibility.*

**Hypotheses** **2c and 2d.** *Relative to a lower level of emotional filial responsibility, the relationship between maternal distress and adolescent anxiety (H2c) and depression (H2d) would be stronger under a higher level of emotional filial responsibility.*

Research Question 3: Does adolescent gender moderate: (a) the direct relationship between maternal distress and children’s mental health problems (indexed by anxiety and depression), (b) the interactive effect between maternal distress and instrumental filial responsibility, and (c) the interactive effect between maternal distress and emotional filial responsibility among Chinese single-mother families experiencing economic disadvantage, respectively?

**Hypotheses** **3a and 3b.** *At higher levels of maternal distress, adolescent anxiety (H3a) and depression (H3b) would be higher for girls than boys.*

**Hypotheses** **3c and 3d.** *At higher levels of maternal distress and instrumental filial responsibility, adolescent anxiety (H3c) and depression (H3d) would be higher for girls than boys.*

**Hypotheses** **3e and 3f.** *At higher levels of maternal distress and emotional filial responsibility, adolescent anxiety (H3e) and depression (H3f) would be higher for girls than boys.*

Research Question 4: Does adolescent age moderate: (a) the direct relationship between maternal distress and children’s mental health (indexed by anxiety and depression), (b) the interactive effect between maternal distress and instrumental filial responsibility, and (c) the interactive effect between maternal distress and emotional filial responsibility among Chinese single-mother families experiencing economic disadvantage, respectively?

**Hypotheses** **4a and 4b.** *At higher levels of maternal distress, adolescent anxiety (H4a) and depression (H4b) would be higher for older adolescents than younger adolescents.*

**Hypotheses** **4c and 4d.** *At higher levels of maternal distress and instrumental filial responsibility, adolescent anxiety (H4c) and depression (H4d) would be higher for older adolescents than younger adolescents.*

**Hypotheses** **4e and 4f.** *At higher levels of maternal distress and emotional filial responsibility, adolescent anxiety (H4e) and depression (H4f) would be higher for older adolescents than younger adolescents.*

## 2. Method

### 2.1. Participants and Procedure

As single-mother families experiencing economic disadvantage were a “hidden” community in Hong Kong to avoid social stigmatization [36], and a complete list of single-mother families was unavailable in Hong Kong, we adopted a purposeful sampling design in the study. A total of 325 mother–child dyads from poor single-mother families were recruited as the respondents. There were three criteria in selecting the respondents: (1) Chinese families facing single motherhood; (2) at least one child aged between 11 and 17; and (3) the monthly household income of the family was lower than the official poverty threshold, i.e., 50% of the median monthly domestic household income in Hong Kong. Those families that received intensive counselling services were excluded in the study.

We invited different non-governmental organizations (NGOs) providing community support programs for single-parent families to participate in the study. Finally, six NGOs, with 19 social service centres across Hong Kong, joined the study. The participants were members of these social service centres who met the inclusion and exclusion criteria. Social workers from the NGOs were given briefings on the selection of service targets as well as procedures on data collection.

There were 325 mother–adolescent dyads of single-mother families participating in the study. From the mothers’ data, the mean age of the mothers was 44.1 (SD = 5.80). Among them, 222 (68.3%) were divorced, 23 (7.1%) were separated, 45 (13.8%) were widowed and 29 (7.1%) reported other status (e.g., unmarried, lost contact, etc.) (6 participants did not respond). A high proportion of mothers had a low educational level, with 183 mothers (56.7%) completing education at junior secondary level or lower, while 113 (34.8%) completed secondary level and 27 (6.5%) had post-secondary level (2 participants did not respond). A majority of families received Comprehensive Social Security Assistance (CSSA) (n = 209, 64.3%), which is a means-tested financial assistance given to poor families. The figures compared favourably with the official statistics of CSSA recipients in Hong Kong [3]. The mean and mode of the number of children in the families was 1.76 (SD = 0.77) and 2, respectively. Regarding the adolescent sample, there were 184 boys (56.6%) and 141 girls (43.3%). The mean age of adolescents was 13.5 (SD = 2.10). For educational level, n = 67 (21.3%) studied in primary schools, n = 182 (56.0%) in junior secondary level, and n = 65 (20.0%) in senior secondary level (n = 11, 3.4% did not respond). A total of 123 (37.8%) were lone children, and 86 (26.5%) were the eldest children in the family.

Data collection was conducted either in the social service centres or at respondents’ homes, subject to the preference of the respondents. Written informed consent of both mothers and adolescents was sought. Mothers were invited to fill out the Mother Questionnaire that contained measures of maternal distress and some demographic characteristics (e.g., age, educational level, number of children, CSSA recipients, etc.), whereas the adolescents were invited to fill out the Adolescent Questionnaire that contained measures of filial responsibilities, mental health attributes and some demographic features (gender, age, educational level, sibling order, etc.) The questionnaire was completed by each participant separately in a self-administered format. The respondents returned the questionnaire either by hand (those that completed the questionnaires at social service centres) or by mail in a sealed envelope provided by the researchers separately. Each respondent would receive a supermarket coupon of HKD 50 (USD 6.4) as compensation for his/her time and effort. The study was approved and monitored by the Human Subjects Ethics Sub-committee of an internationally recognized university.

### 2.2. Measurements

#### 2.2.1. Maternal Distress

The Kessler Psychological Distress Scale (K10) is a self-report measurement that assesses one’s global psychological distress [44]. A sample item is “During that month, how often do you feel nervous?”. Respondents were invited to rate on a 5-point Likert scale (1 = “All of the time” to 5 = “None of the time”). The K10 has been used with Chinese adolescents and showed good reliability [45]. Higher scores of the K10 indicate higher distress. The K10 showed good internal consistency in this study (α = 0.87).

#### 2.2.2. Adolescent Mental Health

Chinese Hospital Anxiety and Depression Scale (HADS-C). Leung et al. [46] translated the Hospital Anxiety and Depression Scale (HADS; [47]) into a Chinese version of HADS (HADS-C) and showed acceptable psychometric properties in a validation study in Hong Kong [48]. There are two dimensions of HADS-C; a 7-item Anxiety Subscale, and a 7-item Depression Subscale. A sample item of the Anxiety Subscale reads “I feel tense or ‘wound up’”, and that of Depression Subscale reads “I have lost interest in my appearance”. Each item was assessed on a 4-point (0, 1, 2, and 3) Likert scale from “0 = not at all” to “3 = most of the time”. Higher mean scores in the Anxiety Subscale and Depression Subscale indicate higher levels of anxiety and depression, respectively. Both subscales showed acceptable reliability of both time points (Anxiety Subscale: α at T1 and T2 = 0.75 and 0.76; Depression Subscale: α at T1 and T2 = 0.69 and 0.67).

#### 2.2.3. Adolescent Filial Responsibilities

The original Filial Responsibility Scale for Youth (FRS-Y) was developed by Jurkovic et al. [49,50], with three subscales: Instrumental Filial Responsibility Subscale (IFRS), Emotional Filial Responsibility Subscale (EFRS) and Perceived Unfairness Subscales (PUS). The measurement was translated into Chinese and then back-translated by two professionals who were fluent in both English and Chinese, and was validated in a sample of 617 Chinese adolescents in Hong Kong [51]. In this study, we used both the IFRS and the EFRS. Each item was rated on a 4-point Likert scale ranging from “Strongly disagree” to “Strongly agree”. The sample item of IFRS reads “I often do the laundry in my family”, and that of EFRS reads “If someone in my family is upset, I try to help in some way”. Higher mean scores of IFRS and EFRS indicate higher levels of instrumental and emotional filial responsibilities, respectively. Both IFRS and EFRS indicated good internal consistency (IFRS: α = 0.81; EFRS: α = 0.87).

### 2.3. Data Analysis

Correlational analyses were performed to examine the relationships between maternal distress, filial responsibilities (instrumental and emotional), adolescent mental health attributes (anxiety and depression) and various socio-demographic characteristics (adolescent gender and age, mother’s educational level, number of children in the family, sibling order, etc.).

In addition, we performed hierarchical multiple regression analyses to test the hypotheses. In testing the moderating effect of instrumental filial responsibility on the relationship between maternal distress and adolescent anxiety, we first entered covariates into the hierarchical regression blocks. Then, the predictor (i.e., maternal distress) and the moderator (i.e., instrumental filial responsibility) were added to the regression equation. Next, maternal distress and instrumental filial responsibility were mean-centred. The interaction term of “maternal distress X instrumental filial responsibility” was computed and added to the regression equation. If the interaction term was significantly related to adolescent anxiety, the moderating effect was supported. Simple slope analyses [52] were performed and plotted graphs were used to illustrate the effects of maternal distress on adolescent anxiety at high levels (1 *SD* higher than the mean) and low levels (1 *SD* lower than the mean) of instrumental filial responsibility.

To examine whether the main effect of maternal distress and the interactive effect of instrumental filial piety and maternal distress on adolescent anxiety varied between adolescent boys and girls, three interaction terms, “maternal distress X adolescent gender”, “instrumental filial responsibility X adolescent gender” and “maternal distress X instrumental filial responsibility X adolescent gender”, were computed and put into the regression equation. If the regression of the interaction term “maternal distress X adolescent gender” on adolescent anxiety was significant, the moderating effect of adolescent gender on the relationship between maternal distress and adolescent anxiety was supported. If the interaction term “maternal distress X instrumental filial responsibility X adolescent gender” on adolescent anxiety was significant, the moderating effect of instrumental filial responsibility in the relationship between maternal distress and adolescent anxiety across adolescent gender was supported. To test the moderating of adolescent age, we created two dummy variables of “younger adolescents” and “older adolescents” using median spilt. Identical procedures were performed to examine the moderating effects of emotional filial responsibility in the relationship between maternal distress and adolescent anxiety and depression, respectively, and whether the main and interactive effects would be different according to adolescent gender and age, respectively.

## 3. Results

For demographic characteristics, correlation analyses showed that mothers’ educational levels were linked to maternal distress. While boys, older adolescents, earlier-born children and those with more siblings reported more adolescent instrumental filial responsibility, older children and later-born children reported more emotional filial responsibility. In addition, earlier-born adolescents exhibited greater anxiety than did later-born adolescents. While maternal distress was positively related to adolescent anxiety and depression, emotional filial responsibility was negatively associated with adolescent depression. The correlation matrix for the variables is listed in Table 1.

**Figure 1:**
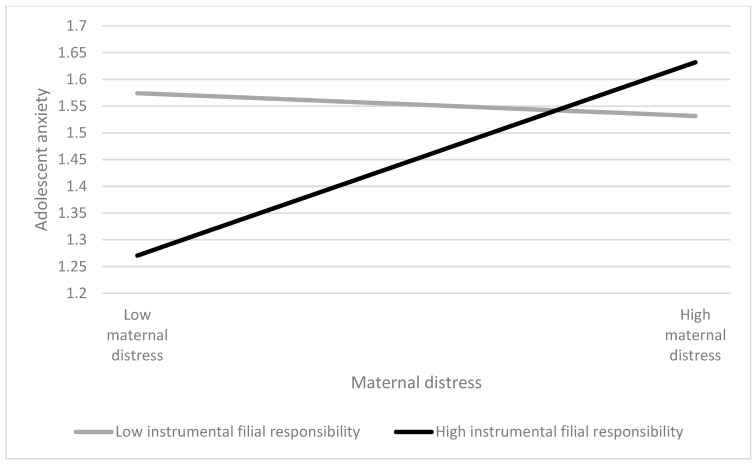
Regression of adolescent anxiety by maternal distress in high and low levels of instrumental filial responsibility.

After controlling for the covariates (adolescent gender and age, sibling birth order, number of children in the family, and mother’s educational level), maternal distress marginally predicted adolescent anxiety, with *β* = 0.11 (*p* < 0.10) (Table 2). H1a was marginally supported. Instrumental filial responsibility moderated the relationship between maternal distress and adolescent anxiety among single-mother families experiencing economic disadvantage, with a significant standardized regression coefficient of adolescent anxiety by “Maternal distress × Instrumental filial responsibility” (*β* = 0.12, *p* < 0.05; Table 2). At a higher level of instrumental filial responsibility, maternal distress was associated with greater adolescent anxiety, with *β* = 0.29 (*p* < 0.01), whereas the association of maternal distress and adolescent anxiety was non-significant at lower levels of instrumental filial responsibility (*β* = −0.03; *p* > 0.05; Table 3) (Figure 1). H2a was supported. Adolescent gender and age neither moderate the direct relationship between maternal distress and adolescent anxiety, nor that regarding instrumental filial responsibility in the association of maternal distress with adolescent anxiety (Table 2).

**Figure 2:**
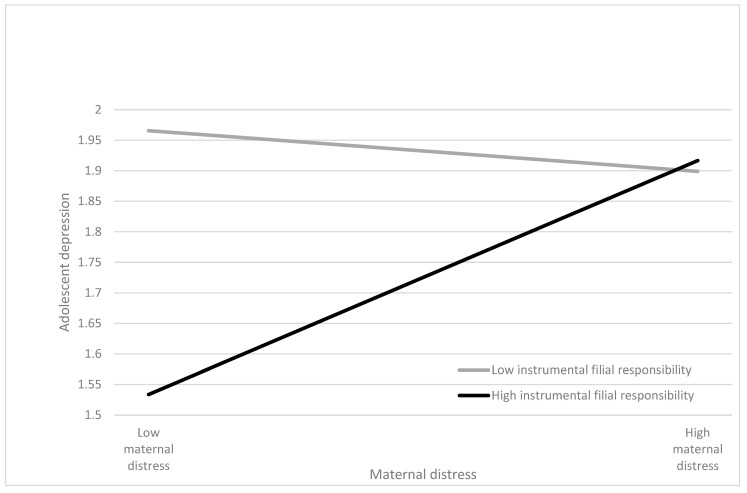
Regression of adolescent depression by maternal distress in high and low levels of instrumental filial responsibility.

Moreover, instrumental filial responsibility moderated the relationship between marital distress and adolescent depression, with a significant standardized regression coefficient of “Maternal distress × Instrumental filial responsibility” on adolescent depression (*β* = 0.13, *p* < 0.05; Table 2). When mothers exhibited greater distress, adolescent children reported more depressive symptoms when they demonstrated higher levels of instrumental filial responsibility (*β* = 0.32, *p* < 0.01; Table 3) than those demonstrating lower levels of instrumental filial responsibility (*β* = −0.06; *p* > 0.05; Table 3) (Figure 2). H2b was supported. Again, adolescent gender and age did not moderate the direct relationship between maternal distress and adolescent depression, or instrumental filial responsibility in the association of maternal distress with adolescent depression (Table 2).

**Figure 3:**
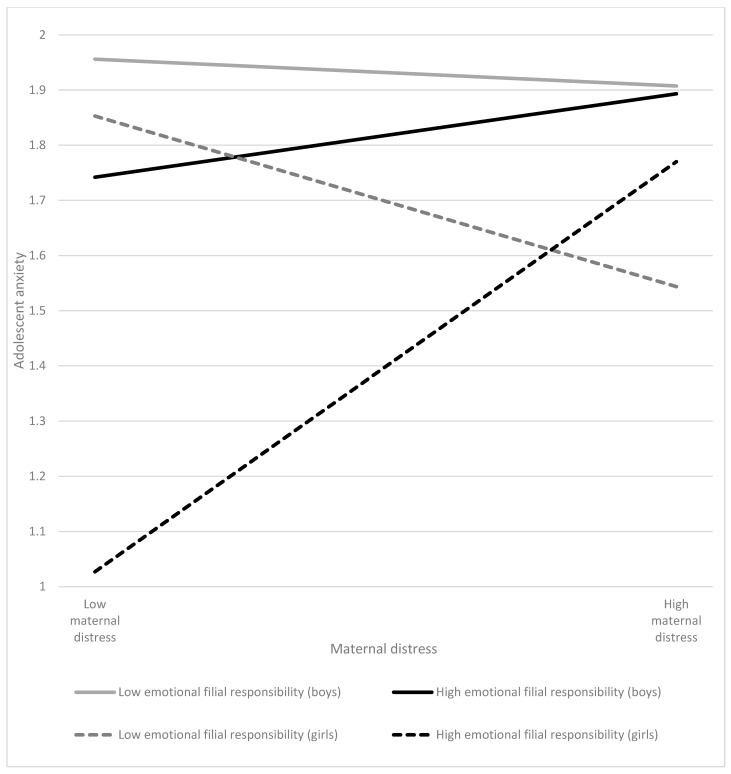
Regression of adolescent anxiety by maternal distress in high and low levels of emotional filial responsibility between adolescent boys and girls.

Regarding emotional filial responsibility as a moderator, emotional filial responsibility moderated the relationship between maternal distress and adolescent anxiety (*β* = 0.16; *p* > 0.01; Table 2). The association of maternal distress with adolescent anxiety was higher when adolescents performed more emotional filial responsibility (*β* = 0.34; *p* < 0.01; Table 3) than in those who performed less (*β* = −0.14; *p* > 0.05; Table 3). In addition, adolescent gender moderated the association of interaction between maternal distress and emotional filial responsibility with adolescent anxiety, with *β* = 0.12 (*p* < 0.05; Table 2). In general, boys were more anxious in single-mother families than were girls. Moreover, at higher levels of maternal distress, girls with higher levels of emotional filial responsibility reported greater anxiety than those having lower levels of emotional filial responsibility. In contrast, the association of maternal distress and adolescents’ anxiety was invariant among adolescent boys regardless of the different levels of emotional filial responsibility they performed for their families (Table 3). Figure 3 showed the associations of maternal distress and adolescent anxiety at higher (+1 *SD*) and lower (−1 *SD*) levels of emotional filial responsibility. However, emotional filial responsibility did not moderate the relationship between maternal distress and adolescent depression (Table 2).

## 4. Discussion

This study examined the associations of maternal distress with adolescent mental health problems in poor Chinese single-mother families in Hong Kong, and the moderating effects of filial responsibilities (indexed by instrumental filial responsibility and emotional filial responsibility) in the relationships. The findings showed some support of the Family Stress Model that maternal distress was associated with adolescent anxiety and depression in poor single-mother families [8]. Moreover, instrumental filial responsibility moderated the relationship between maternal distress and adolescent anxiety and depression. At lower levels of maternal distress, adolescent anxiety and depression was at the lowest level when adolescents performed instrumental filial responsibility. According to Role Identity Theory [23], adolescents’ performance of household chores and care-giving to their siblings demonstrated their commitment towards the family [17]. Their assistance in housework may ease the workload of mothers and facilitate better family functioning, which contributes to the positive family adaptation process in the face of single motherhood and poverty [38].

However, when maternal distress increased, adolescent children performing more instrumental filial responsibility exhibited higher levels of anxiety and depression. There are three possibilities that account for the findings. First, according to Family Systems Theory [21], parentification involves an enmeshed relationship between mothers and adolescents [22]. Adolescents who performed more filial responsibility may be easily tangled with their mother’s negative emotions, i.e., adolescents who are more filial may be more anxious and depressed when they share the grief and pain of their mother. Second, when mothers exhibit distress, they may ignore the contributions of their children in performing the filial roles. The failure of maternal recognition and mattering may increase adolescent anxiety and depression [53]. Third, an increase of maternal distress may imply greater demands from mothers and other family members on adolescents’ commitment in fulfilling household chores and caregiving for younger siblings, if mothers fail to fulfil their responsibilities at home. These additional demands further suppress the age-appropriate needs of the children, which are detrimental to their wellbeing [19,28,29].

The findings further indicated that emotional filial responsibility moderated the associations of maternal distress with adolescent anxiety. Furthermore, the moderating role of emotional filial responsibility was found to be different between adolescent boys and girls. In general, boys were more anxious in single-mother families than were girls. In Chinese culture, as males take up a dominant role to protect the family [33], the absence of the father implies that the son needs to shoulder the responsibility to take care of the family and be involved in the decision-making process (i.e., the surrogate father), which may create stress and anxiety for adolescent boys. Moreover, the results showed that greater maternal distress was related to greater anxiety among adolescent girls who perform many emotional filial responsibilities. On the other hand, when girls performed less emotional filial responsibility, the association of maternal distress with girls’ anxiety was negative. However, the association of maternal distress and adolescent anxiety was non-significant for boys, regardless of different levels of emotional filial responsibility. When performing emotional caregiving duties, girls are highly involved in their mothers’ distress and become enmeshed in the mother–daughter relationship [39], which may heighten their anxiety level. In contrast, when girls take up less emotional filial responsibility, they can have a better self-differentiation from their mother and get less involved in their mothers’ emotion, which is better for their wellbeing [54]. For adolescent boys, the association of maternal distress with adolescent anxiety was invariant regardless of the emotional caregiving they offer. One possibility is that males are more task-oriented and pay less attention to emotions in their coping [55], making them less sensitive to maternal distress in fulfilling emotional filial responsibility. The other possibility is that the “surrogate father” role has been demanding for them [56,57]. Hence, an increase in emotional filial responsibility may not change the relationship between maternal distress and boys’ wellbeing. As studies on the father’s role taking of adolescent boys in single-mother families are severely lacking, more research in this area is suggested.

There are several theoretical and practical implications of the present findings. First, the study suggests that parentification does happen in single-mother families experiencing economic disadvantage [26,29]. Facing maternal distress, adolescent children taking up more instrumental and emotional filial responsibility reported poorer mental health when compared with those who take up less filial responsibilities. Though the study was conducted in the Chinese context, the findings echo some studies performed in the U.S. where adolescents displayed more distress and anxiety, more disciplinary problems and poorer academic performance arising from instrumental and emotional caregiving (30, 58). These findings alarm researchers and family practitioners regarding the needs and situations of adolescent children and their mothers in these underprivileged families. As adolescents need to perform household chores and take care of the emotional needs of their mother and siblings, over and beyond the age-appropriate developmental tasks that they can accomplish, this would lead to stress and anxiety. Unfortunately, poor single-mother families are hidden groups to avoid social labels aligned with single motherhood and poverty [36]. More resources and services should be given to these families. Family counselling is necessary to relieve the emotions of mothers and other family members, and reorganize the family roles and functions so that the adolescent children are not troubled by these additional family responsibilities. Moreover, more tangible resources such as a family aide service would be helpful to relieve the burdens of single mothers and children due to family distress and poverty.

Furthermore, the present study showed that adolescent boys exhibited consistently high levels of anxiety in poor single mother families, and adolescent girls who performed more emotional filial responsibility reported increasing anxiety when their mothers expressed greater distress. These findings contribute to the social science literature by highlighting gender differences in the interactive effects of emotional caregiving and maternal distress on adolescent anxiety, which has been inadequately explored in family studies. Moreover, family scholars and practitioners may need to pay more attention to gender difference in family roles taken by adolescent children in poor single-mother families and their relationship with adolescent stress. The sons may need to take up the role of a “surrogate father” [56,57], and the daughters may need to provide emotional caregiving to their distressed mother, which may generate great stress and anxiety for them. Family practitioners and youth workers may need to examine the underlying family roles of adolescents in the family, and provide appropriate support for the adolescents and their families.

Despite the pioneer findings in a Chinese context, there are several limitations of the study. First, as the study is cross-sectional, it has the intrinsic limitation of drawing causal relationship between the variables. Hence, there is a need to conduct longitudinal studies in future. Second, non-random sampling via recruitment from social service units was adopted in the study due to the incomplete list of poor single-mother families, as well as difficulties in identifying these families. As a result, those families that did not receive social service assistance were excluded in the study. Adolescent children in these families might be the more vulnerable, as they did not receive any assistance from the community, and their mental health might be more affected by filial obligations to take care of their families. Third, the study was conducted in Hong Kong. Though the findings support some studies conducted in Western societies [30,58], there is a need to replicate the study in other Chinese communities in Mainland China, Taiwan and elsewhere.

## 5. Conclusions

The present study is pioneering in examining the moderating role of instrumental and emotional filial responsibilities in the relationship between maternal distress and adolescent mental health among Chinese single-mother families experiencing economic disadvantage. The study revealed that instrumental and emotional filial responsibilities intensified the relationship between maternal distress and poorer adolescent mental health in single-mother families experiencing economic disadvantage. Parentification may occur when adolescent children take up more instrumental and emotional filial responsibilities in helping their distressed mothers, which was linked to poorer mental health in adolescents. In response to McLanahan and Sandefur [56]’s questions on “what helps” and “what hurts” in adolescents growing up in single-mother families, this study provides insightful insights into the roles of filial responsibilities in the association of maternal distress and adolescent wellbeing in Chinese contexts.

## Figures and Tables

**Table 1:** Correlations of the measuring variables.

	Mean	SD	1	2	3	4	5	6	7	8	9
1. Maternal distress	2.54	0.89	1.00								
2. Instrumental filial responsibilities	1.89	0.78	0.06	1.00							
3. Emotional filial responsibilities	2.30	0.61	−0.01	0.37 ***	1.00						
4. Anxiety	2.09	0.56	0.12 *	0.00	−0.08	1.00					
5. Depression	1.97	0.54	0.13 *	−0.07	−0.19 **	0.48 ***	1.00				
6. Adolescent gender (boys = −1; girls = 1)	N.A.	N.A.	0.03	0.13 *	0.06	0.11	0.02	1.00			
7. Adolescent age	13.53	2.10	−0.02	0.13 *	0.13 *	0.08	0.02	−0.06	1.00		
8. Sibling birth order	N.A.	N.A.	0.01	0.34 ***	−0.12 **	0.12 *	0.05	−0.06	0.03	1.00	
9. No. of children in the family	1.76	0.77	0.06	0.44 ***	−0.05	0.11	0.04	0.10	0.15 **	0.72 ***	1.00
10. Mother’s educational level	N.A.	N.A.	0.12 *	0.04	−0.04	0.00	−0.01	0.03	−0.03	−0.09	−0.06

* *p* < 0.05, ** *p* < 0.01, *** *p* < 0.001.

**Table 2:** Regression of adolescent mental health by instrumental and emotional filial responsibility in the context of maternal distress.

Moderator		Anxiety			Depression		
		b	SE	β	B	SE	β
Instrumental filial responsibility	Step 1						
	Gender of adolescents	0.16	0.07	0.15 *	0.04	0.06	0.04
	Age of adolescents	0.03	0.02	0.10	0.01	0.02	0.02
	Sibling birth order	0.06	0.05	0.11 ^†^	0.04	0.05	0.07
	No. of children	0.01	0.06	0.02	−0.02	0.06	−0.03
	Mother’s education	0	0.04	0.00			
	Step 2						
	Maternal distress	0.07	0.04	0.11 ^†^	0.07	0.03	0.11 ^†^
	Instrumental filial responsibility	−0.06	0.05	−0.08	−0.12	0.05	−0.16 *
	Step 3						
	Maternal distress × Instrumental filial responsibility	0.10	0.05	0.12 *	0.11	0.05	0.13 *
	Gender as a moderator:						
	Step 4						
	Maternal distress × Gender	0.07	0.07	0.06	0.14	0.07	0.11 ^†^
	Instrumental filial responsibility × Gender	0.01	0.09	0.01	0.14	0.08	0.10 ^†^
	Step 5						
	Maternal distress × Instrumental filial responsibility × Gender	0.19	0.10	0.11 ^†^	0.00	0.10	0.00
	Age as a moderator						
	Step 4						
	Maternal distress × Age	0.02	0.02	0.08	0.00	0.02	−0.01
	Instrumental filial responsibility × Age	0.01	0.02	0.04	0.10	0.05	0.12 ^†^
	Step 5						
	Maternal distress × Instrumental filial responsibility × Age	−0.01	0.02	−0.04	0.03	0.02	0.09
Emotional filial responsibility	Step 1						
	Gender of adolescents	0.16	0.07	0.14 *	0.04	0.07	0.04
	Age of adolescents	0.03	0.02	0.10†	0.01	0.02	0.03
	Sibling birth order	0.06	0.05	0.11	0.03	0.05	0.06
	No. of children	0.01	0.06	0.01	−0.03	0.06	−0.04
	Mother’s education	0.00	0.04	0.00	−0.01	0.04	−0.01
	Step 2						
	Maternal distress	0.07	0.04	0.11 ^†^	0.06	0.04	0.09
	Emotional filial responsibility	−0.09	0.05	−0.09	−0.21	0.05	−0.23 ***
	Step 3						
	Emotional filial responsibility × Maternal distress	0.15	0.05	0.16 **	0.08	0.05	0.09
	Gender as a moderator:						
	Step 4						
	Maternal distress × Gender	0.07	0.07	0.06	0.02	0.11	0.01
	Emotional filial responsibility × Gender	−0.08	0.11	−0.04	0.07	0.05	0.08
	Step 5						
	Maternal distress × Emotional filial responsibility × Gender	0.23	0.11	0.12 *	0.06	0.11	0.03
	Age as a moderator						
	Step 4						
	Maternal distress × Age	0.02	0.02	0.08	0.03	0.02	0.11 ^†^
	Emotional filial responsibility × Age	0.02	0.03	0.05	−0.03	0.03	−0.08
	Step 5						
	Maternal distress × Emotional filial responsibility × Age	−0.01	0.02	−0.03	−0.01	0.02	−0.03

^†^*p* < 0.10, * *p* < 0.05, ** *p* < 0.01, *** *p* < 0.001.

**Table 3:** Simple slope analyses of the relationship between maternal distress and adolescent mental health with adolescent gender and filial responsibilities as moderators.

Moderator		Predictor	Regression Coefficient (*β*)		
			Overall	Boys	Girls
			Anxiety		
Instrumental filial responsibility	Higher level (+1 *SD*)	Maternal distress	0.29 **	N.A.	N.A.
	Lower level (−1 *SD*)		−0.03	N.A.	N.A.
Emotional filial responsibility	Higher level (+1 *SD*)	Maternal distress	0.34 **	0.13	0.56 ***
	Lower level (−1 *SD*)		−0.14	−0.04	−0.23
			Depression		
Instrumental filial responsibility	Higher level (+1 *SD*)	Maternal distress	0.32 **	N.A.	N.A.
	Lower level (−1 *SD*)		−0.06	N.A.	N.A.

** *p* < 0.01, *** *p* < 0.001.

## Data Availability

Datasets generated for this research are available from the corresponding author upon reasonable request.
